# Hospital and Institutional News

**Published:** 1917-10-20

**Authors:** 


### HOSPITAL AND INSTITUTIONAL NEWS.

### SIR ALFRED KEOGH, G.C.B., M.D., F.R.C.S.

Sir Alfred Keogh visited the Royal College of Surgeons, Lincoln's Inn Fields, last week to open an Exhibition of War Office Specimens of Military Injuries which has been arranged by the College staff and is temporarily housed in the museum. On this occasion the diploma of honorary fellowship of the Royal College of Surgeons was conferred upon Sir Alfred by Surgeon-General Sir George Makins, who paid tribute to the brilliant career of the Director-General in the medical service of the Army. After acknowledging the high honour paid to him, Sir Alfred Keogh called attention to the important character of the exhibition, and said that in the collection of the exhibits three names stood out—those of Dr. Morley Fletcher, Sir John Bland Sutton, and Captain G. B. Bartlett, R.A.M.C. Specimens were included sent in by the Medical Services of the Armies of the Oversea Dominions as well as by the Royal Army Medical Corps, and it was hoped that eventually these would go to the Dominions to form the nuclei of pathological museums there. It was also hoped that out of the superabundance of materials which this war would supply, local museums would be created in some of the military hospitals. The exhibition is comprehensive in character and should attract widespread attention. There is a large group of such things as gas helmets, metal helmets and respirators, and the purely pathological exhibits comprise objects of interest in connection with previous wars as well as the present war. Included are a number of models obtained by Sir Victor Horsley from his experiments to demonstrate the explosive effects of high-velocity bullets.

### ORTHOPÆDIC HOSPITALS FOR SOLDIERS

King Manuel of Portugal is indefatigable in his activities on behalf of military orthopædic hospitals, and the curative workshops which are being established in association with them up and down the country owe their initiation in a large measure to his energetic advocacy and support. On Oct. 9, he conducted the members of the Allied Conference on Pensions, which has been sitting in London, over the Orthopædic Hospital at Shepherd's Bush, which he referred to as the "Father of the Family," while that recently opened at Golder's Green he called "The Baby." The members of the Conference later paid visits to other hospitals and training institutions. After a visit to Newcastle to address a meeting for launching the fund for the new orthopædic establishment which is being built in connection with the Newcastle Infirmary, King Manuel is expected at Cardiff to open the curative workshops at the orthopædic centre for Wales, the Welsh Metropolitan War Hospital at Whitchurch.

### THE AMERICAN AMBASSADOR AT LEEDS.

The second extension carried out at the 2nd Northern General Hospital at Beckett's Park, Leeds, since the outbreak of the war was opened by Dr. Page, the American Ambassador, on Wednesday in last week (October 10). The ceremony took place in the central hall of the institution, and, owing to the limited accommodation available, the tickets of invitation were restricted to persons connected in some way or another with the movement. The gallery was occupied by the nurses. The Lord Mayor, in sketching the wonderful growth of the hospital, mentioned that more than £40,000 had been subscribed by Leeds and its surroundings for the two extensions. The hospital, now equipped to receive 1,800 patients, is devoting itself wholly to the orthopædic treatment of wounded soldiers. Colonel Littlewood is chief administrator of the hospital, which includes on its staff surgeons from the United States.

### THE CARDIFF HOSPITAL DEFICIT.

A striking appeal to the whole community to provide further funds for King Edward VII.'s Hospital, Cardiff, was made by Col. E. M. Bruce-Vaughan at the October meeting of the board of management. He pointed out that the institution will be faced with a deficit of £8,000 or £9,000 at the end of the year, and the policy of the board of management was to try to prevent it. He mentioned that he had been associated with the hospital for twenty years, during which period the debt had always been the bugbear, and that only in 1914 a debt was paid off which had risen to £30,000. The extensions in progress were proved to be absolutely necessary by their waiting-list of over 1,000 patients. No hospital could remain stationary if the population of the district it served was increasing, and they must institute in Cardiff, as had been done in London and some important provincial towns, a quinquennial appeal. If they were able to carry through their present scheme 180 beds would be added, making the total 500. The estimated capital expenditure of the work was £150,000, towards which there had been paid or promised £71,631, leaving £78,369 to obtain. When the whole of the 180 beds were in use they would require an addition of £15,000 to their annual income, making the total income needed every year £35,000 in round figures. He suggested that efforts should be put forth to frame a great and comprehensive scheme and to enlist the support of the Lord Mayor, the Chairman of the County Council, the President of the Chamber of Commerce, the Chairman of the Coalowners' Association, and the Chairman of the Shipowners' Association. If they tackled the problem in a bold, statesmanlike manner, as had been done in Liverpool, Manchester, and Birmingham, the needs of their hospital, as well as of the smaller hospitals in the district, would be met.

### THE POWER OF THE PENNY.

There were few money-raising ideas, Col. Bruce-Vaughan said, which were not carried out at King Edward VII.'s Hospital. Their invested capital of £140,000 was kept intact, and was constantly being added to. The Aberdare League took street collections. Then there also was Alexandra Day. The Cardiff Hospital Society organised the penny-a-week scheme, and there was a system by which each patient in the hospital paid for his food if he could afford to do so. There were, roughly, 225,000 colliers and other workmen employed in the South Wales coalfield, and if they could but induce each of them to subscribe one penny a week that would produce a sum of £48,750 per annum, and if the masters and coal-owners would add, say, half the amount of the men's contributions, £24,375, there would be a sum of £73,125 a year, which would not only give the hospital a sufficient income for all its needs, but would provide a handsome sum for research work in their medical school. In concluding, Col. Bruce-Vaughan said that if some such scheme were not adopted their magnificent voluntary hospitals would become chargeable to the rates, which, in his opinion, would be a great calamity, as it would dry up broad streams of human sympathy that flow at present.

### A MEDICAL BOARD'S ACTION.

There was a sharp difference of opinion between medical and lay members of the board of management of King Edward VII.'s Hospital, Cardiff, at the monthly meeting on October 10. In recognition of the valuable services he has rendered to the hospital during the past seventeen years, the board were asked by Col. Bruce-Vaughan to elect Mr. Geary Grant, an assistant surgeon, to the honorary staff. Members of the medical board objected, on the ground that the election was inopportune, against precedent, and against the machinery which had been set up for the careful working of the institution. The proposal was seconded, however, by Dr. R. J. Smith, the ex-Lord Mayor, and passed by a majority of one; but Major Maclean entered a protest against the vote, and when the Chairman put it as a substantive motion he and his medical colleagues in khaki declined in a body to vote in favour of recommending the election committee to make the appointment after an adverse report from the medical board.

### THE BIRMINGHAM SATURDAY INSTITUTIONS.

It is pleasant to recur to the steady growth of the group of institutions which bud, as it were, from the healthy stock of the Birmingham Hospital Saturday Fund. The Ministry of Munitions has allowed the men's block of the home at Droitwich, known as Highfield, to receive the addition of a wing for women, and this is now on the eve of completion. The home is designed for the treatment of rheumatism, and if the work done since the spring in the men's block had not been a success, the Ministry of Munitions, whose heart is probably a shell, would hardly have allowed an extension, though the need for it seemed incontestable to those on the spot. There will be eventually sixty beds, and the cost is expected to be £13,500, of which the odd figures above £10,000 remain to be raised. Kynochs and the Birmingham Small Arms Co. have been subscribers, and indeed the responsibility for financing the whole scheme was from the start accepted by the working classes. Here, again, is an example of the power of class or sectional organisation in the raising of income. And in this, as we emphasise on every possible occasion, lies a solution of the difficulty of maintenance which rises before those hospital managers who are anxious to do away with the disgrace of a long waiting-list by building fresh wards, which yet they fear cannot be maintained without debt. What the working classes have done, the professional and lower middle classes can do also. The way is plain, the method clear, the means at hand—for those who have the energy to employ them.

### BUMBLE IN UNIFORM.

We are glad to see that the editor of *Truth* is still pegging away to remove the red tape which strangles the unhappy patients in the Royal Naval Hospitals of Plymouth and Chatham. Here is a field for his energies which is unexceptionable; here an honourable channel for his experience to navigate. The public may probably take his word for it that the leave is limited in absurd ways, which only an official in uniform with nothing to do (which is the curse of the Services in peace-time) would have the folly to invent. Bumble is by no means peculiar to the town hall or the municipal parlour or the old vestry. He has strutted the quarter-deck before now. He has waddled on parade. He is everywhere, in fact, where bounce is tolerated as part of a professional make-up. His method, as *Truth* amusingly points out, is to treat his victims like "a troublesome child," and if the victim is neither troublesome nor a child, Bumble is the more delighted to worry him. In short, he is no person for either Service, and the Directors-General of each Medical Service should so act as to make a thought of themselves and this old humbug together impossible and extravagant.

### ORTHOPÆDICS SIXTY YEARS AGO.

Hospital history so weaves itself around the lives of men and women that the good hospital histories are a record of manners almost as much as a record of fact. An example is the letter lately received by Mr. Edward Ansell at a recent meeting of the Royal Orthopædic and Spinal Hospital, Birmingham, which the wife of one of the former honorary surgeons had sent. Over sixty years ago, Mrs. E. M. Warden wrote, when Dr. Warden was on the staff, the institution was known as one for "the Relief of Bodily Deformities." It then consisted of a couple of rooms in Great Charles Street. Its first move was to Newhall Street, from which it went to the present house. Additions were made. The Marquis of Hertford became president—a great step in those days, and he received Queen Victoria's permission to have the hospital called by its present title. That was in the 'sixties. It was for nearly half a century that Dr. Warden acted as honorary surgeon before he retired to Devonshire. But the history of the hospital is that of its men of energy, and the present chairman, Mr. George A. Bryan, is looking forward to a new scheme of enlargement.

### THE ABSENCE OF STANDARD IN SMALL WAR HOSPITALS.

Anyone with first-hand experience of the military hospitals knows that there is a curious difference of standard in different institutions. The big military hospitals are on the whole astonishingly well administered; but the smaller and remoter institutions, the so-called semi-convalescent hospitals (not the homes where actual convalescence is passed), vary enormously. There is, in fact, no general standard of efficiency at all. Such efficiency as there is entirely depends on the officer or matron in charge. It would not be difficult, by an inspection of the smaller hospitals, to collect instances which would give a serious impression of inefficiency. But there is no one to do it. The inspectors, even the old hospital men who have accepted the task, are tied by red tape, and having deposited their report the facts are buried, even though improvement is secured. Even a trained administrator with a free hand would find it extremely hard to tell the truth about the smaller military hospitals without conveying a wrong impression. For such defects as exist, even the grave ones, are as haphazard as the successes. To tell the story of one patient who had been neglected in some way might lead the public to suppose that every patient in the man's ward was also treated carelessly. The reverse is often true. A is neglected. B is waited on hand and foot. A ward is sometimes under-staffed, sometimes not. The whole thing is disorder. That is the approximate truth. How can it be remedied without careful and responsible inspection?

### PREPARING FOR AIR-RAIDS IN HOSPITALS.

The recent air-raids over London have naturally caused some alarm, especially amongst patients occupying some of the top wards in the large hospitals and infirmaries. At the Lambeth Infirmary Dr. Lionel Baly, the medical superintendent, has made arrangements so that only patients who can easily be removed to the basement are now accommodated in these wards. This applies to all wards except the male phthisical wards, where the patients have expressed a strong desire to be allowed to remain in their own wards. Dr. Baly states that these arrangements add considerably to the difficulty of staffing the wards, since some wards, of necessity, contain an unduly large number of patients requiring a large amount of nursing, whereas in others the work is very light. Other hospitals and infirmaries have made similar plans, and even if the transference of the patients may not materially increase their safety, it will often add greatly to their mental relief, an important point in many cases. At the Bethnal Green Infirmary beds have been put in the basement, and the Guardians have especially thanked the staff for the excellent manner in which they dealt with the situation. At Leyton the Council has for the time being closed the isolation hospital and arranged for the transfer of patients to one of the institutions of the Metropolitan Asylums Board.

### SUNDERLAND SCHOOLS MEDICAL OFFICER.

A debate in which some acute feeling was shown occurred at a recent meeting of the Sunderland Corporation on the question of the position of Dr. E. S. Murray, the acting schools medical officer, in relation to Army service. Dr. Murray is twenty-seven years of age, he is unmarried, and he has been passed as fit for general service. Some members urged that he ought to be released for military work, but the Education Committee's reply was that hitherto the local medical committee had been unable to arrange for the discharge of Dr. Murray's duties by anyone else. Medical men, however, had written offering to do the work by rota, and their communication was referred to the Education Committee. A proposal to increase Dr. Murray's salary from £300 to £400 a year was ruled out of order.

### HAVE GENERAL PRACTITIONERS' FEES RISEN?

"There is one form of 'profiteering' to which I have not hitherto seen any public reference, and that is the recent practice of certain medical practitioners of charging double fees for professional attendance." Thus the writer of a letter to the *Morning Post*, which does not appear to have evoked much comment. We certainly have no reason to suppose that as a general rule practitioners have put up their fees, and we do not believe, except in perhaps a very few instances, fees have been doubled. There is nothing ethically wrong in a certain increase, especially in the case of the general practitioner who dispenses his own prescriptions, where his outlay in drugs, dressings, and bottles must be considerably higher than in the time when he arranged the amount of remuneration he should receive. We believe that as a rule the doctor stands in the same position as the architect, the barrister, or any other professional man, and as the Government official or person with a fixed salary. His expenditure in every direction has much increased, while the rate under which personal profit is regulated has remained the same as formerly.

### MEAT INSPECTION.

There are few things more important for the health of a community than the provision of good food, and therefore the inspection of meat is of the greatest value, but statistics show that different inspectors have very different standards, therefore a discussion which is to be held at a meeting of the Royal Sanitary Institute should prove of use. The discussion is on "Standards of Meat Inspection under War and other Conditions." It is to be held on November 9 at Newcastle-on-Tyne, and it will be opened by Mr. Thomas Parker, F.R.C.V.S., who is Veterinary Officer and Inspector of Pro visions at Newcastle.

### THE VALUE OF TUBERCULIN.

Dr. E. H. Snell, the medical officer of health for Coventry, in his annual report says there is no doubt that tuberculin is a valuable asset in the treatment of surgical tuberculosis and is of some use in pulmonary cases. Its apparent failure in the latter type of case may be due to the fact that the disease is generally too advanced before inoculation is commenced. He treated last year sixteen cases of lung tuberculosis. Eight were in an advanced stage when he took charge of the dispensary, eight were early cases, and on the whole he says they did fairly well. In three cases the disease apparently became arrested and treatment was discontinued. Two cases of glands in the neck received tuberculin injections, and in both cases the glands rapidly disappeared. The results of injections of tuberculin depend on many factors: on the form of tuberculin employed, on the dose administered, on the situation of the disease and its stage, and on the presence or absence of other organisms. Opinions on the subject differ widely, and this is mainly because the treatment can vary so greatly.

### OPHTHALMIA NEONATORUM.

Whitehall has great faith in the expansive capacities of the Metropolitan Asylums Board and never hesitates to ask that authority to undertake new duties. Now the Local Government Board is asking it to make provision for hospital treatment of certain cases of ophthalmia neonatorum, suggesting that this can best be undertaken by the Asylums Board. It is mentioned that it would suffice if at the outset two hospitals were provided or two special departments added to existing hospitals, one for North and the other for South London, at each of which about twenty-five cases could be accommodated. The matter is important and urgent, for this disease when unchecked is the greatest cause of blindness, and its early energetic treatment will lead in most cases to a complete cure.

### DEPENDANTS OF SOLDIERS IN RATE-AIDED INSTITUTIONS.

The Lambeth Board of Guardians are obtaining the support of the great majority of the London Boards of Guardians in their opposition to the action of the Army Council in stopping the allowances to dependants of soldiers who are forced to seek medical assistance in rate-aided institutions. The matter has been mentioned in several recent issues of The Hospital, but the action taken by the Bermondsey Board of Guardians is one which may result in some reflections. They have left it to the discretion of their collector to come to terms with the dependants when admitted to their institutions as to the sum that should be deducted from the separation allowances to meet the bare cost of maintenance, the remainder of the allowance being left for the support of the children, if any, or the upkeep of the home. One case was brought to their notice at the last meeting by Mr. J. Marriott. A woman, the wife of a soldier, unfortunately had to be removed to an asylum. She had two children, who were left in the care of their grandmother, a poor person in receipt of outdoor relief. Evidently acting under the instructions of the board, the grandmother received out of the 28s. allowance allotted to her daughter one week 6s. and the next 7s., though the rent of the rooms occupied by her daughter was 6s. 6d. a week. Inquiries are to be made into the case, but if the facts are true it would appear that, as Dr. Lionel Baly, the superintendent of the Lambeth Infirmary, said, the dependants of soldiers are in a much worse plight in case of sickness than ordinary civilians. There is only one way to stop these scandals, and that is for the Army Council not to withdraw the allowances in these cases, and for Boards of Guardians to refrain from taking a penny-piece from the dependants.

### SAILORS' AND SOLDIERS' HOME.

At Bridlington, on October 9, the new St. John Home for Pensioned Sailors and Soldiers was opened; it may be followed by the foundation of several other such Homes in the town. The institution, which will accommodate thirty men, is situated on the Marine Drive, commanding a splendid view over the South Bay, and is in every way excellently provided internally. The honorary medical superintendent is Dr. A. Holmes Field, and Mrs. Field is discharging the duties of hon. lady superintendent. They and Mr. F. W. Lendis, chairman of the house committee, are to be congratulated on the efficient lines in which the establishment is planned.

### MORE BEDS FOR SALFORD?

Of extension schemes, save for the future, we can rarely read nowadays, but it is refreshing in their absence to hear of considered plans and the purchase of property. Looking ahead, which is supposed to be characteristic of Manchester notwithstanding the muddle over the old infirmary site —looking ahead is the name for a policy undertaken at Salford, where the Royal Hospital has purchased a site in the rear of the present buildings, which we hope will do something to remove the scandalous shortage of beds from which Manchester, in common with less ambitious cities, suffers. The sketch plans, we trust, are being considered and thought out now. We shall be interested to see how they "pan out."

### AN OFFER WHICH HAS MADE HISTORY.

By the regretted death of Mr. Percy L. Baker, Leicester's Mayor Elect, the Leicester Cripples' Guild has lost a devoted and generous friend whose numerous acts of kindness and service to the members have been of that personal kind which the public knows not of, but which live long in the memories of the recipients. In recent years Mr. Baker's services were freely given to provide for the recreation of wounded soldiers. It was due largely to him that the Leicestershire Automobile Club organised annually a series of motor outings and entertainments which have been enjoyed by thousands of wounded sailors and soldiers. His greatest service to the wounded, however, because its influence extended to every war hospital throughout the country, arose from his offer to the Leicester War Hospitals Committee to give a substantial parcel of tobacco and cigarettes to the wounded "if it could be freed from Customs duty." This challenge became the origin of negotiations and representations through influential sources, with a result that early in 1915 the Treasury consented to waive claims to duty in respect of all gift supplies of tobacco and cigarettes and other dutiable goods for the use of wounded sailors and soldiers in every recognised war hospital in the United Kingdom. Mr. Baker was a member of the Town Council for some years. He also held a seat on the Board of Governors of the Leicester Royal Infirmary.

### "THE NATIONAL FOOD JOURNAL."

If, as we have suggested it should be, the fortnightly journal of the Food Controller is easily procurable by the public, it should do much to clear up obscure points in the Orders and Supplementary Orders which issue with increasing rapidity from headquarters. The journal is at the same time a help and a warning; prominence is given to prosecutions for contravention of the Food Orders, and in addition to the expounding and elucidation of the different decrees, correspondents are invited to put their difficulties before the editor, who replies, where the point is of general interest, in a special column. We learn of people being fined for wrongfully obtaining sugar, and for wasting bread or giving it to animals. If some modern Rip Van Winkle were to wake up and read these paragraphs he would think that the world had gone topsy-turvy. As an instance of the extensive nature of the machinery of the Ministry of Food, it is noted that a correspondent is advised to write to the Ministry giving the name of a fishmonger who does not sell red herrings! The second number of the journal contains a long list of wholesale maximum prices which should be very useful to the institutional caterer.

### THE SANATORIUM TREATMENT COLLAPSE.

Endeavours are being made to call a Conference of Local Authorities to consider the position of consumptives who are no longer able, though insured, to secure sanatorium treatment. The London Insurance Committee honestly admits that it can no longer fulfil its obligations towards the insured consumptives. The Lewisham Council has, in this connection had a report from its Medical Officer of Health, who quotes instances to prove how completely the sanatorium benefits have broken down and refers to the danger caused to the public health by the presence amongst the population of advanced cases of consumption which cannot now be isolated. The Lewisham Council considers the existing state of affairs so serious that it thinks an effort should be made with a view to reorganising the whole system and grappling with the problem, from the point of view of both insured and non-insured persons.

### CONSUMPTION AT GAINSBOROUGH.

The people of Gainsborough are much alarmed by the spread of consumption in the town, as revealed in the special report recently presented by Dr. Hackett, the M.O.H. The matter is regarded in so serious a light by the friendly societies that they have placed the medical officer's figures in the hands of Mr. R. O. Griffith, a well-known Lancashire actuary, and solicited his advice concerning the position of the local societies. Mr. Griffith states that the condition of affairs is very serious, as one-seventh of the Gainsborough death-rate is due to consumption, one person in every fifty-nine of the population being affected, as against one in every 116 in 1916. The mortality from tuberculosis is two to every 1,000 living. Mr. Griffith advises energetic action by the friendly societies, particularly by such as continue benefits throughout sickness, and suggests that the attention of Sir Edward Cornwall, head of the National Insurance Commissioners, should be drawn to the state of affairs, with a view to a recommendation by him to the Local Government Board for assistance. The Friendly Societies' Council at Gainsborough have asked the Urban Council to take prompt action to meet this grave danger to the community.

### CURIOUS PATRIOTISM.

Says the *South African Medical Record* (Cape Town) speaking editorially, "We have little or nothing to do with the merits or demerits of this particular war, or of the particular parties to it. The only fact we fix on to is that it is a war in which the constitutional authorities of the Union of South Africa have decided to take part, which, being so, imposes upon every citizen of that Union the paramount duty of assisting those constitutional authorities, by every means in his power, to win that war." Is this the best the editor of the *Record* can do in the way of patriotism? Can he not, apart from his duty as a citizen, work up any feeling of enthusiasm for the aims of the Allies, dissociated as they are from any possible charge of self-interest, and based as they are on the wish to protect the essential principle of the right of the individual to continue his lawful pursuits without molestation? There is something very Hunnish in the sentiment of blind obedience to a Government or military autocracy, apart from "the merits or demerits" of the cause.

### WAR BONUSES FOR ASYLUM WORKERS.

The vexed question of West Riding Asylum workers' wages has been settled by the withdrawal of the Asylum Board's resolution of August 28, awarding 8s. a week increase to all members of the non-resident staff. As the application of this resolution was found to be full of anomalies and difficulties, they have instead decided to give war bonuses on a generous scale. The new plan was adopted at a special meeting of the Board, and came into force as from the first of the present month. It will continue in force until three months after the end of the war, and is to apply to all members of the staff who are serving with H.M. Forces. An additional war bonus of 5s. per week, with an additional 6d. a week for each child under fourteen years of age, is given to all employees living out, excepting the medical superintendents, assistant medical officers, the solicitor, clerk, architect, treasurer, chaplains and visiting ministers, nurses, and farm labourers; an additional 4s. is given to those who live in by day and sleep out by night (nurses excepted), with 6d. per child, and an additional 2s. to those who live in and sleep in, including the nurses who are excluded from participating in the benefits before mentioned.

### MRS. ATKINS WRITES AGAIN.

The following is a further selection of letters from Mrs. Atkins to the medical and other Army officers to whom she looks, as a soldier's dependant, for the payment of an allowance or pension. We noted last week a note of crescendo in the batch then printed. This crescendo becomes a fortissimo in the sequence, though, as we then pointed out, the letters come from different hands. They are united in their object. The word "it" almost invariably means the money due, and Mrs. Atkins, like her "Tommy," is a multiple personality. The eighth letter reads: "I am expecting to be confined next month, will you please let me know what I am to do about it." The ninth: "My Bill has been put in charge of a spittoon, shall I get any more money." The tenth is almost severe: "Will you please send my money as early as possible, as I am walking around—like a bloody pauper and oblige." The eleventh is a pathetic threat: "Unless I receive my husband's pay at once I shall be compelled to lead an immortal life." The twelfth: "In answer to your letter I have given birth to twins, hoping this will be satisfactory." The thirteenth: "When I received your letter I was ill in bed with happendideti." It will be very useful now." The last should touch even a soldier's conscience, and spur the Minister of Pensions to greater efforts. It runs: "Just a few lines to say that owing to your delay in sending my money we have not a morsel of food in the house. Hoping you are the same." We join in her hope, for an official of the Charity Organisation type is often a person with a pigeon-hole instead of a heart.

### THE FIRST ANNUAL SUBSCRIBER.

After three months' experience of maternity and child-welfare work, Dudley is to have a maternity hospital. The site has been given by Alderman G. Bean, whose plan for devoting his estate, known as The Firs, for some public purpose has now taken the above direction. Mr. Bean wisely hoped, when making his gift, that the Town Council would not be niggardly. In fact, the bedsteads and bedding were promised the same day; while the proposed hospital gained its first annual subscriber in Mr. T. W. Tanfield, who promised, with great public spirit and practical sense, a sum of £25 a year for five years. Child-welfare schemes have often done nothing except advertise their promoters. But at Dudley there is the proverbial exception which illustrates the ordinary rule.

### RESULTS OF BABY WEEK.

The Baby Week campaign, though in some respects not altogether beyond criticism, has certainly had its good results. Due in part to the great advertisement given to such schemes, infant-welfare centres are now being opened in this country at the rate of twenty a month. The detailed accomplishments of the recent campaign have now been gathered together, and a report in book form is soon to be issued. Here is one of the typical claims:—"Owing to the Baby Week Campaign the ill-effects of bad sanitation have been realised in Plymouth, and a committee has been appointed to deal with it." The movement, moreover, is spreading throughout the world. "Our literature is being used now in France, and America has asked for the use of our Motherhood film, and similar requests have been received from South Africa, Mexico, and New Zealand."

### LINCOLN MOTHERCRAFT SCHOOL.

A mothercraft school is to be established at Lincoln under municipal auspices. At a recent meeting of the City Council the Health Committee recommended the acceptance of an offer by two ladies to provide £1,500 for the establishment of a mothercraft school and an infants' clinic, subject to the condition that the premises to be converted should become the property of the Corporation, in whom the control and management should be vested. There was some little opposition to the scheme, one member declaring that it would involve expenditure equal to a twopenny rate, that the upkeep would be £400 to £500 a year, and that the spending of £700 or £800 on structural alterations could not be justified. The Health Committee's recommendation was, however, adopted, and we think that money could hardly be better spent.

### THIS WEEK'S DRUG MARKET.

A distinctly quiet tone is noticeable in the drug market; and although values are, on the whole, well maintained, there are few actual changes of first-class importance. A drug which is attracting attention just now is eucalyptus oil; this being the principal consuming season the demand is considerable, and the price has been steadily advancing for some little time, and it seems most probable that it will rise still further. One of the reasons of this advance is that many steamers refuse to carry the oil except as deck cargo, and this attitude is readily understood when one considers the enormous damage that could be done by a leaking package of eucalyptus oil to a cargo of wheat. Other causes of the advance are the shortage of labour in the producing centres and the high cost of insurance. A small business continues to be done in quinine, and the price still has an upward tendency. Epsom salts are in small supply and considerable demand, with the result that the high price is well maintained. There is no further change in the value of bromides, but the demand continues good. Cream of tartar is again dearer, and the scarcity is unrelieved. Jalap is rather dearer. The scarcity of sugar of milk is still very acute. A good business is reported in glycerophosphates, and prices are well maintained. In synthetic drugs there are few changes of note; phenacetin, salicylic acid, and sodium salicylate are in rather better supply, and the values are not quite so firmly maintained.

